# Ohio College of Dental Surgery

**Published:** 1882-05

**Authors:** 


### OHIO COLLEGE OF DENTAL SURGERY.

The thirty-sixth annual commencement.

The number of matriculates for the session was sixty-eight.

The degree of D.D.S. was conferred on the following persons by James Leslie, D.D.S., President of the Board of Trustees.

| NAME. | RESIDENCE. |
|---|---|
| Edwin L. Ashton | Michigan. |
| Harry F. Anshutz | Ohio. |
| Clarence W. Bard | Pennsylvania. |
| Alexis Bertrand | France. |
| Chas. F. Braffet | Ohio. |
| Wm. A. Bettman | Ohio. |
| A. F. Bowman | Ohio. |
| Edward E. Ball | Ohio. |
| J. E. Barriklow | Ohio. |
| C. G. Burgin | Kentucky. |
| James W. Dennis | Ohio. |
| Charles N. Dann | Ohio. |
| Charles Dappen | Germany. |
| George W. Dengler | Kentucky. |
| C. L. Franks | Ohio. |
| Robert Goebel | Illinois. |
| Wm. D. Green | Illinois. |
| Christian A. Herr | Ohio. |
| Chas. G. Junkermann | Ohio. |
| Cyrus T. King | Ohio, |
| W. C. Kerns | Ohio. |
| J. Walter Mann | Illinois. |
| J. H. Maust | Pennsylvania. |
| J. S. Mardis | Pennsylvania. |
| Chas. S. Ogborne | Indiana. |
| Jas. S. Perkins | Wisconsin. |
| Legrand B. Perry | Indiana. |
| Al. O. Ross | Ohio. |
| Frank D. Rice | Kentucky. |
| W. H. Smith | Ohio. |
| William H. Todd | Ohio. |
| Townsend J. Thomas | Iowa. |
| Wm. M. Williams | Ohio. |
| Forest O. Welker | Colorado. |
| Wesley L. Williams | Michigan. |

